# A SARS-CoV-2 antigen rapid diagnostic test for resource limited settings

**DOI:** 10.1038/s41598-021-02128-y

**Published:** 2021-11-26

**Authors:** Erica Frew, Douglas Roberts, Shelly Barry, Matthew Holden, Amanda Restell Mand, Emily Mitsock, Enqing Tan, Wei Yu, Johan Skog

**Affiliations:** 1 Bio-Techne, Devens, MA USA; 2 Exosome Diagnostics, A Bio-Techne Brand, Waltham, MA USA

**Keywords:** Diseases, Health care

## Abstract

Severe Acute Respiratory Syndrome Coronavirus 2 (SARS-CoV-2) is the causative agent of COVID-19 disease. RT-qPCR has been the primary method of diagnosis; however, the required infrastructure is lacking in many developing countries and the virus has remained a global challenge. More inexpensive and immediate test methods are required to facilitate local, regional, and national management strategies to re-open world economies. Here we have developed a SARS-CoV-2 antigen test in an inexpensive lateral flow format to generate a chromatographic result identifying the presence of the SARS-CoV-2 antigen, and thus an active infection, within a patient anterior nares swab sample. Our 15-min test requires no equipment or laboratory infrastructure to administer with a limit of detection of 2.0 × 10^2^ TCID_50_/mL and 87.5% sensitivity, 100% specificity when tested against 40 known positive and 40 known negative patient samples established by a validated RT-qPCR test.

## Introduction

As of June 2021, the World Health Organization (WHO) has tallied over 3.7 million deaths caused by the SARS-CoV-2 virus^1^. This virus has spread globally to all corners of the planet, highlighting healthcare inequities, and impacting the most vulnerable populations. The virus dispersion across remote settings still presents a barrier to testing access^2,3^.

Quantitative real-time reverse transcription polymerase chain reaction (RT-qPCR) is the gold standard test method for confirmation of SARS-CoV-2 infection^4^. Despite its superior clinical performance, RT-qPCR is challenging to implement in resource-limited settings due to its expensive reagents, supply chain challenges, longer time to result, and requirements for either a central laboratory environment or sophisticated instrumentation. Although not as sensitive as RT-qPCR, rapid diagnostic tests (RDTs) based on lateral flow technology are inexpensive and allow for patient testing in non-laboratory settings. RDTs have been successfully implemented in the control of HIV and malaria^5^, and while not as useful for diagnosing asymptomatic patients with low viral load^6^, RDTs are quickly becoming an essential tool in the SARS-CoV-2 testing arsenal to keep world economies open^7^. RDTs can be applied more often, and it is important that we shift focus from a high analytical sensitivity (the ability to detect low viral copy numbers in a sample) to the more relevant metric of a test’s sensitivity to detect infections in a population. A test that is not available or accessible for frequent use is not as likely to be effective as a surveillance regimen to limit viral spread^8^. Even in developed countries like the United States, the Center for Disease Control and Prevention (CDC) estimated that there were 10 times as many COVID-19 cases than reported^9^. This means despite the high analytical sensitivity of RT-qPCR testing, when used as a surveillance testing regimen it has at best a 10% sensitivity to detect the circulating infections in the population^8^.

Culture-positive patient specimens which indicate potentially contagious viral levels are generally not found beyond day 9 after the onset of SARS-CoV-2 symptoms, with most transmission occurring before day 5^10,11^. Rapid identification of symptomatic patients allows for immediate implementation of isolation and other efforts to arrest transmission of the virus. The WHO recommends that SARS-CoV-2 antigen RDTs meeting minimum performance requirements of ≥ 80% sensitivity and ≥ 97% specificity compared to a RT-qPCR reference assay could be used to diagnose SARS-CoV-2 infection within the first 5–7 days following the onset of symptoms^12^. In settings where RT-qPCR is unavailable, or where prolonged time to result makes clinical utility a challenge, the ability to offer an inexpensive alternative that can be run at the point of care and deliver immediate results is essential. Regions with lower capacity for RT-qPCR could utilize such an RDT to screen symptomatic patient samples, employing the use of PCR for only the RDT negative samples to save time and resources.

We have developed a SARS-CoV-2 antigen RDT that is instrument free and easy-to-use at the point of care with 15 min testing time intended to be used in concert with approved RT-qPCR methods.

## Methods

### SARS-CoV-2 antigen RDT device design and manufacture

**Figure 1:**
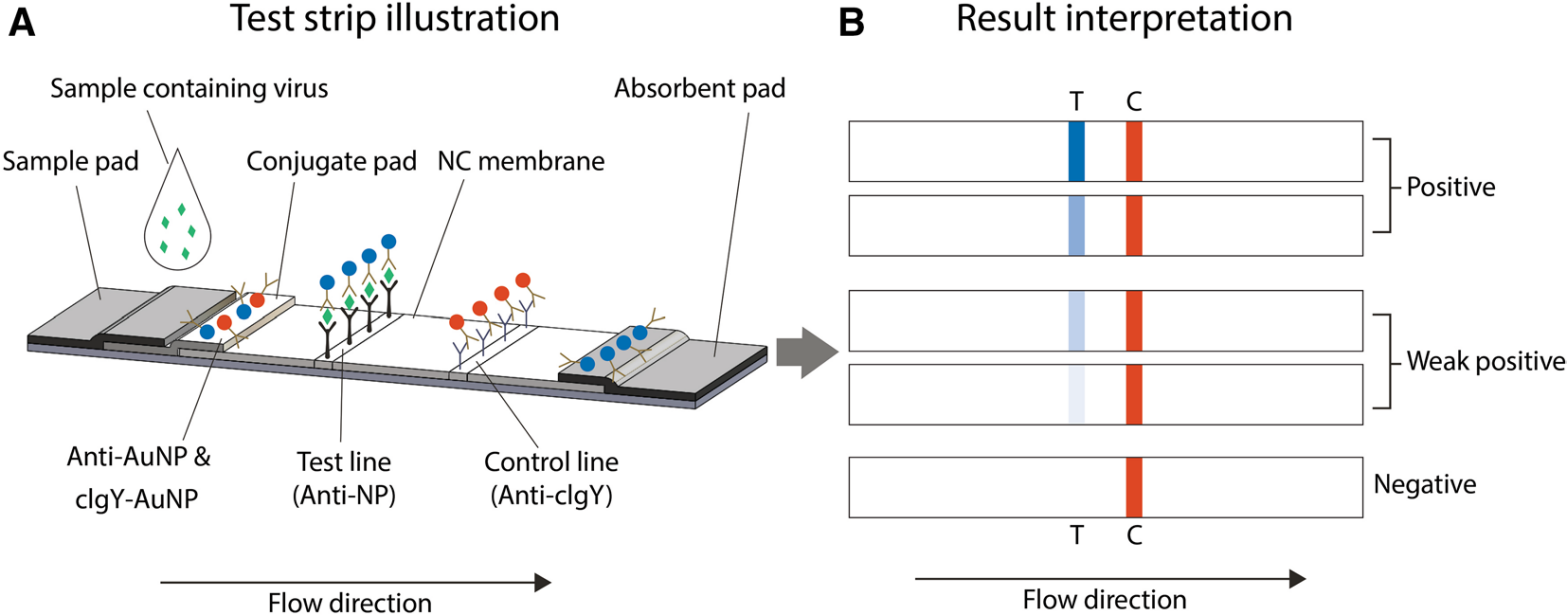
Lateral flow test strip design and assay principle (**A**). Mouse anti-nucleocapsid protein antibody conjugated to 150 nm gold particles (anti-AuNP) are for mouse anti-nucleocapsid protein (anti-NP) test line detection. Chicken IgY antibodies conjugated to 40 nm gold particles (cIgY-AuNP) are for goat anti-chicken IgY (anti-cIgY) control line detection. Visual result interpretation (**B**). The red colored control line (C) must be present for a test to be valid. The control line serves to monitor the proper liquid flow and that the bio-reagents of the test device are active. Presence of a visible blue test line (T) indicates the sample is positive for the SARS-CoV-2 virus. Absence of a blue test line indicates the sample is negative or below the detection limit of the test. The test cassette can also be read using a cassette reader for quantitative evaluation.

Our SARS-CoV-2 antigen RDT utilizes lateral flow immunoassay technology. Each test strip consists of a plastic backing card, sample pad, conjugate pad with the detection particles, nitrocellulose detection membrane with immobilized antibodies, and wicking pad. The test strip design, assay principle, and visual result interpretation are illustrated in Fig. 1.

Nitrocellulose membrane cards are prepared by adhering 25 mm × 301 mm wide nitrocellulose membrane (Sartorius Stedim Biotech) to 60 mm × 301 mm vinyl backing cards (DCN Diagnostics) using a Matrix 2210 Universal Laminator (Kinematic Automation). A test line consisting of mouse anti-nucleocapsid protein antibody solution (Meridian Bioscience), and control line consisting of goat anti-chicken IgY solution (Bio-Techne), are printed onto the nitrocellulose membrane using low contact pressure nozzles on an IsoFlow Reagent Dispenser (Imagene Technology). Printed membrane cards are dried at 37 °C and stored in sealed foil pouches with silica desiccant until further processing.

To prepare the conjugate pad, mouse anti-nucleocapsid protein antibody (Meridian Bioscience) is conjugated to 150 nm gold carboxyl nanoparticles and chicken IgY (Bio-Techne) is conjugated to 40 nm gold carboxyl nanoparticles as control as previously described^13^. Both conjugation protocols utilize standard EDC chemistry as recommended by the particle manufacturer (nanoComposix). Test and control particles are combined in a drying buffer containing sugars, stabilizing proteins, and surfactant and sprayed onto 8 mm × 300 mm glass fiber conjugate pads (Millipore) using an atomizer nozzle on an IsoFlow Reagent Dispenser. Sprayed conjugate pads are dried at 37 °C and stored in sealed foil pouches with silica desiccant at ambient room temperature until further processing.

Printed nitrocellulose membrane cards are assembled with sprayed and dried conjugate pads, 17 mm × 300 mm cellulose fiber sample pads (Millipore), and 20 mm × 300 mm cellulose fiber wicking pads (Millipore) using a Matrix 2210 Universal Laminator. Pads are overlapped as depicted in Fig. 1A to allow for capillary flow from the sample pad, through the conjugate pad, up the nitrocellulose membrane, and finally into the wicking pad.

Fully assembled cards are cut into 3.8 mm test strips using a Matrix 2360 Programmable Shear (Kinematic Automation) and assembled into plastic test cassettes (Venus-lab) using a Closure-1 press (A-point Technologies). Individual cassettes and 1 g silica desiccant (ULINE) are sealed into foil pouches (Labels Inc.) to prevent moisture from degrading antibody components.

**Figure 2:**
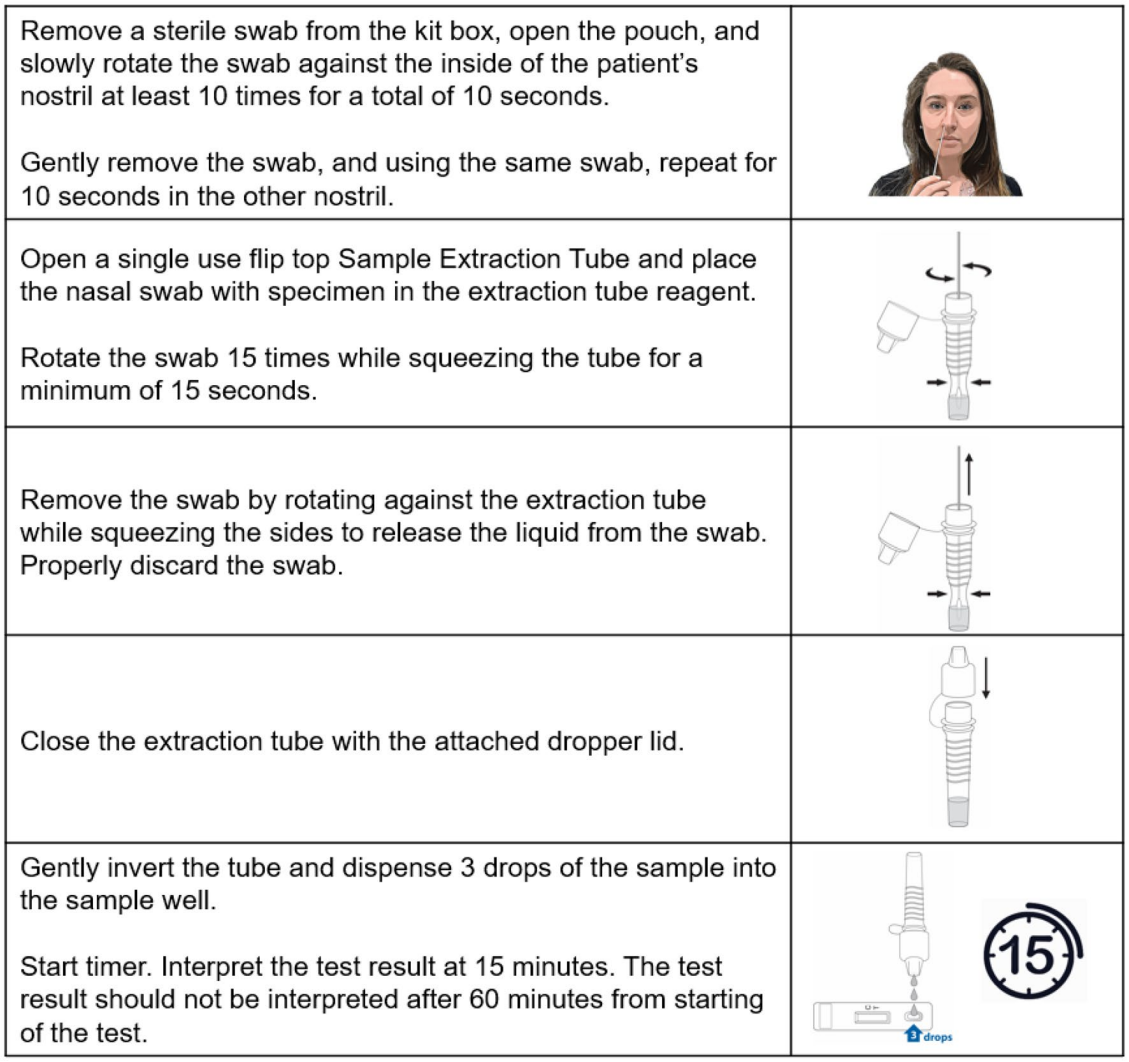
Pictorial description of SARS-CoV-2 antigen RDT intended sample testing procedure.

The current Test Kit contains 25 test cassettes sealed in foil pouches with desiccant, 25 sterile nasal swabs, 25 sample extraction tubes with 400 µL of sample extraction buffer each, 1 positive and 1 negative control swab, a package insert, and a quick reference instruction card. Intended use will allow for testing and result interpretation at the point of care, where a patient nasal swab sample is collected and immediately extracted into the buffer of the sample extraction tube. The tube is capped with the attached dropper tip and 3 drops are applied to the RDT cassette; signal is interpreted visually after 15 min as depicted in Fig. 2 (consent to publish was obtained from the person whose image is included in Fig. 2). The sample extraction buffer consists of a phosphate buffered saline solution with casein and mouse IgG as blocking agents, Brij 35 as surfactant and lysis agent, and sodium azide as preservative.

Recombinant Sf 21 (baculovirus)-derived SARS-CoV-2 nucleocapsid protein Met1-Ala419 (Bio-Techne) is used as quality control throughout the test manufacturing process. The SARS-CoV-2 antigen rapid test is designed to be interpreted visually; however, for this study we also employed a hand-held RDS-2500 reader (Detekt Biomedical) to provide objective, quantitative data in support of our development activities and throughout the manufacturing process, as well as to register the visual interpretation of results by the user.

### SARS-CoV-2 reference sample

Heat inactivated SARS-CoV-2 isolate USA-WA1/2020 was acquired through BEI Resources. The virus isolate was inactivated by heating to 65 °C for 30 min. The pre-inactivation titer was 1.6 × 10^5^ TCID_50_ per mL and the genome copy number was evaluated using BioRad QX200 Droplet Digital PCR to be 3.75 × 10^8^ genome equivalents/mL per the manufacturer’s certificate of analysis.

### Clinical sample acquisition and characterization

Nasal swab samples were collected after swabbing both anterior nares for approximately 10 s. Swabs were inserted into a transport tube containing saline solution and shipped over-night at ambient temperature to the ExosomeDx CLIA laboratory. All samples used in this study were previously collected, de-identified remnants from diagnostic testing at the ExosomeDx CLIA laboratory. Samples were collected and de-identified in accordance with the HIPAA Privacy Rule and determined exempt from IRB review as they do not meet the definition of human subject as defined in US federal regulation 45 CFR 46.102 (WCG IRB, formerly Western Institutional Review Board). All methods associated with these samples were performed in accordance with the relevant guidelines and regulations. The viral load in each sample was assessed by the ExoDx COVID-19 RT-qPCR Test (authorized by FDA under an Emergency Use Authorization (EUA) and validated by Exosome Diagnostics CLIA laboratory). De-identified saline swab samples were stored at -80 °C after RT-qPCR testing until further analysis.

### Sample preparation for clinical performance testing

For clinical performance studies, as samples were not collected directly into our RDT kit sample extraction buffer tube, a 10X formulation of the sample extraction buffer was prepared and added directly into the saline sample matrix to mimic our intended sample-extraction buffer concentration ratio. De-identified samples were removed from the − 80 °C freezer and allowed to thaw and equilibrate to room temperature in a sample processing hood. Once thawed, the samples were briefly vortexed and centrifuged for 5 min at 4000 rpm. For each sample, 10 µL of 10X extraction buffer was added to 90 µL of nasal swab saline sample, pipetting up and down to mix. 85 µL of this resulting sample mix was dispensed via pipette into the sample well of the RDT cassette.

Additional nasal swab samples collected into saline were mixed 1:1 directly into the kit sample extraction tubes (400 µL of saline collected sample into 400 µL 1X extraction buffer) to better mimic the RDT intended use per the test kit instructions. Three drops (approximately 85 µL) were added to the sample port of the RDT cassette.

In each case a timer was started upon sample addition and results were interpreted after 15 min.

### SARS-CoV-2 antigen RDT device interpretation

After the 15-min incubation, the cassette was visually assessed and determined to be positive or negative. Positive results were indicated by the presence of a blue test line of any intensity at the “T” location and the presence of a red control line at the “C” location. Negative results were indicated by a lack of blue test line of any intensity at the “T” location and the presence of a red control line at the “C” location. An invalid test would have no red control line at the “C” location; there were no invalid RDT cassettes observed out of > 200 cassettes tested, therefore in the studies reported here, the invalid test rate was 0%. Immediately after visual inspection cassettes were inserted into the RDS-2500 reader and read to measure the signal intensity at both the test and control line locations.

## Results

### SARS-CoV-2 RDT lot consistency

**Figure 3:**
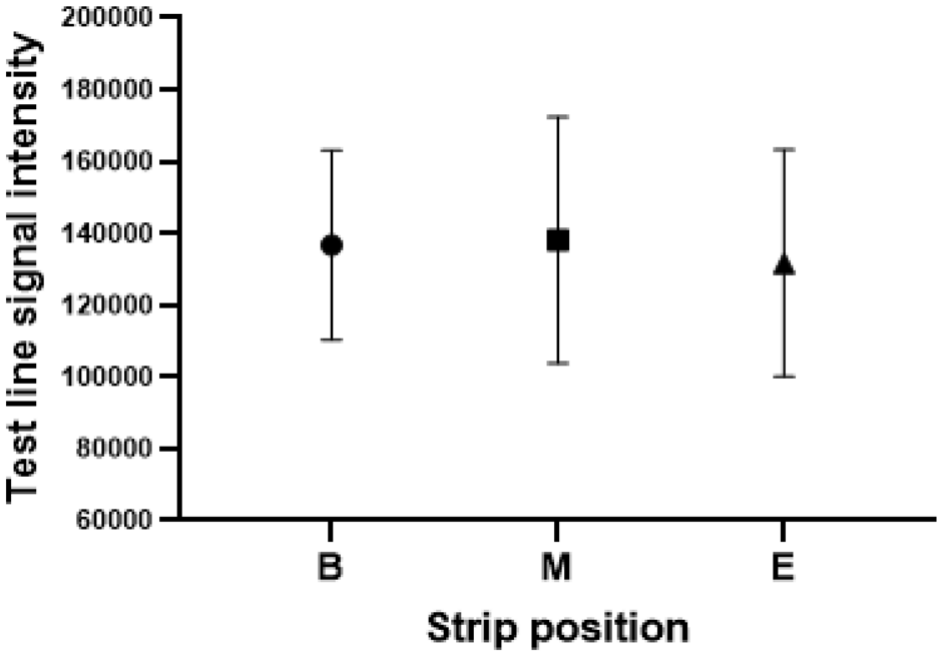
SARS-CoV-2 RDT cassette intra-lot reproducibility. Interval plot of RDS-2500 test line signal intensity vs. beginning (B), middle (M), or end (E) strip location using 2.5 ng/mL recombinant nucleocapsid antigen in sample extraction buffer. No significant difference in performance was observed across the entire lot of test devices.

One lot of 2750 test cassettes were prepared from a total of 38 printed and assembled cards, with approximately 72 test strips produced from each card. Each test strip was assembled into a cassette and sealed into a foil pouch with a silica desiccant packet. To evaluate the intra-lot consistency, test cassettes assembled with strips collected from the beginning, middle, and end of each even-numbered card were collected. Recombinant nucleocapsid antigen at 2.5 ng/mL in sample extraction buffer was chosen to target 2X the LOD signal on the RDS-2500 reader; 85 µL was dispensed into the sample port of each cassette. After 15 min each cassette was visually inspected and run on an RDS-2500 reader and intensity values recorded for both test and control lines; an interval plot of test line signal intensity vs. strip location is depicted in Fig. 3. Results were analyzed by one-way ANOVA in Minitab with a significance level of α = 0.05; test line intensity *p* = 0.800 and control line *p* = 0.270 demonstrated no significant difference in performance across the entire lot of test devices.

### Linearity and limit of detection of the RDT device

Linearity and Limit of Detection (LOD) studies utilized the guidelines outlined by the U.S. Food and Drug Administration Coronavirus Disease 2019 (COVID-19) Emergency Use Authorizations for Medical Devices Antigen Template for Test Developers, October 26, 2020 version^14^. Heat inactivated SARS-CoV-2 virus (BEI Resources, isolate USA0WA 1/2020) was diluted into a pool of 10 individual RT-qPCR SARS-CoV-2 negative nasal samples collected into saline.

**Figure 4:**
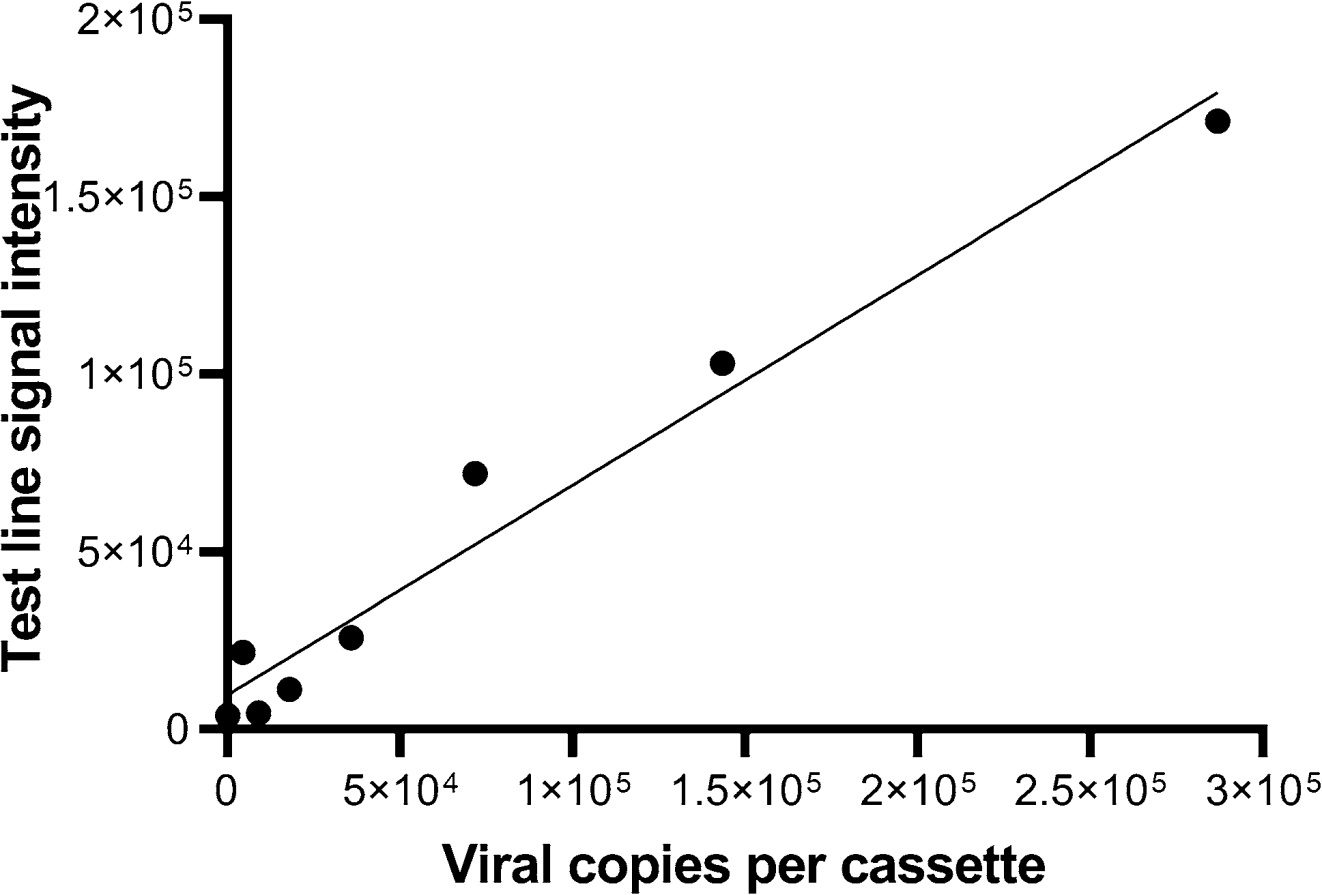
Detection of SARS-CoV-2 antigen is proportional to viral input. RDS-2500 reader quantified test line intensity of serially diluted SARS-CoV-2 heat-inactivated virus spiked into negative nasal swab sample matrix. Test line signal counts were correlated to viral copy input with a resultant R^2^ of 0.9684.

Linearity was evaluated by testing serial dilutions of inactivated SARS-CoV-2 virus in pooled negative nasal swab samples and the intensity of the test line was evaluated by visual inspection (Table 1) as well as quantified using the RDS-2500 reader (Fig. 4).

**Table 1:** Linearity study summary.

Viral copies/RDT	TCID_50_/mL	Test line result (+/−)	Control line result (+/−)
2.86 × 10^5^	1.6 × 10^3^	+	+
1.43 × 10^5^	8.0 × 10^2^	+	+
7.17 × 10^4^	4.0 × 10^2^	+	+
**3.59 × 10**^**4**^	**2.0 × 10**^**2**^	** + **	** + **
1.79 × 10^4^	1.0 × 10^2^	−	+
8.96 × 10^3^	50	−	+
0	0	−	+

The dilution in **bold** (3.59 x 104 viral copies/RDT, 2.0 x 102 TCID50/mL) was the lowest detectable dilution in the series.

Visually interpreted test results of heat inactivated reference SARS-CoV-2 virus dilutions into pooled negative nasal swab sample (n = 2 per dilution). All samples with 1.79 × 10^4^ viral copies or less per RDT cassette as well as the zero-virus negative control did not generate a visible test line and were interpreted as negative. All test cassettes have a visible red control line, indicating all tests were valid.

Following visual inspection, RDT cassettes were read in the RDS-2500 reader to quantify line intensity. Test line signal counts were correlated to viral copy input. Figure 4 demonstrates a linear response between the amount of viral material applied to the cassette and the amount of specific SARS-CoV-2 signal detected at the test line. The correlation coefficient for the resultant best fit line demonstrated an R^2^ of 0.9684, indicating an acceptable dose–response for the N-antigen detection assay.

The FDA EUA guidance defines LOD as the lowest concentration at which 19 of 20 (95%) replicates are positive^14^. Limit of Detection Robustness was evaluated by running n = 20 of the dilutions above, at, and below the presumed LOD of 3.59 × 10^4^ viral copies per test cassette; results are summarized in Table 2 below and the assay image of each RDT cassette can be found in Supplemental Fig. 1. The LOD was verified using inactivated virus spiked into pooled negative nasal swab samples and found to be 3.59 × 10^4^ genomic viral copies or 200 TCID_50_/mL per RDT cassette (Table 2).

**Table 2:** LOD robustness using heat-inactivated virus in pooled negative swab samples.

Viral copies/RDT	Positive test line	Positive control line
1.79 × 10^4^	0/10	10/10
**3.59 × 10**^**4**^	**20/20**	**20/20**
7.17 × 10^4^	20/20	20/20

The dilution in **bold** (3.59 x 104 viral copies/RDT, 2.0 x 102 TCID50/mL) was the lowest detectable dilution in the series.

SARS-CoV-2 RDT cassette replicates of both 7.17 × 10^4^ viral copies/RDT and 3.59 × 10^4^ viral copies/RDT had all 20/20 cassettes with positive test lines by visual inspection. For the 10 RDT cassettes to which 1.79 × 10^4^ viral copies/RDT was applied, all 10/10 test lines were negative by visual inspection. The lowest level of virus where at least 95% of the replicates were detected was 3.59 × 10^4^ viral copies/RDT.

**Figure 5:**
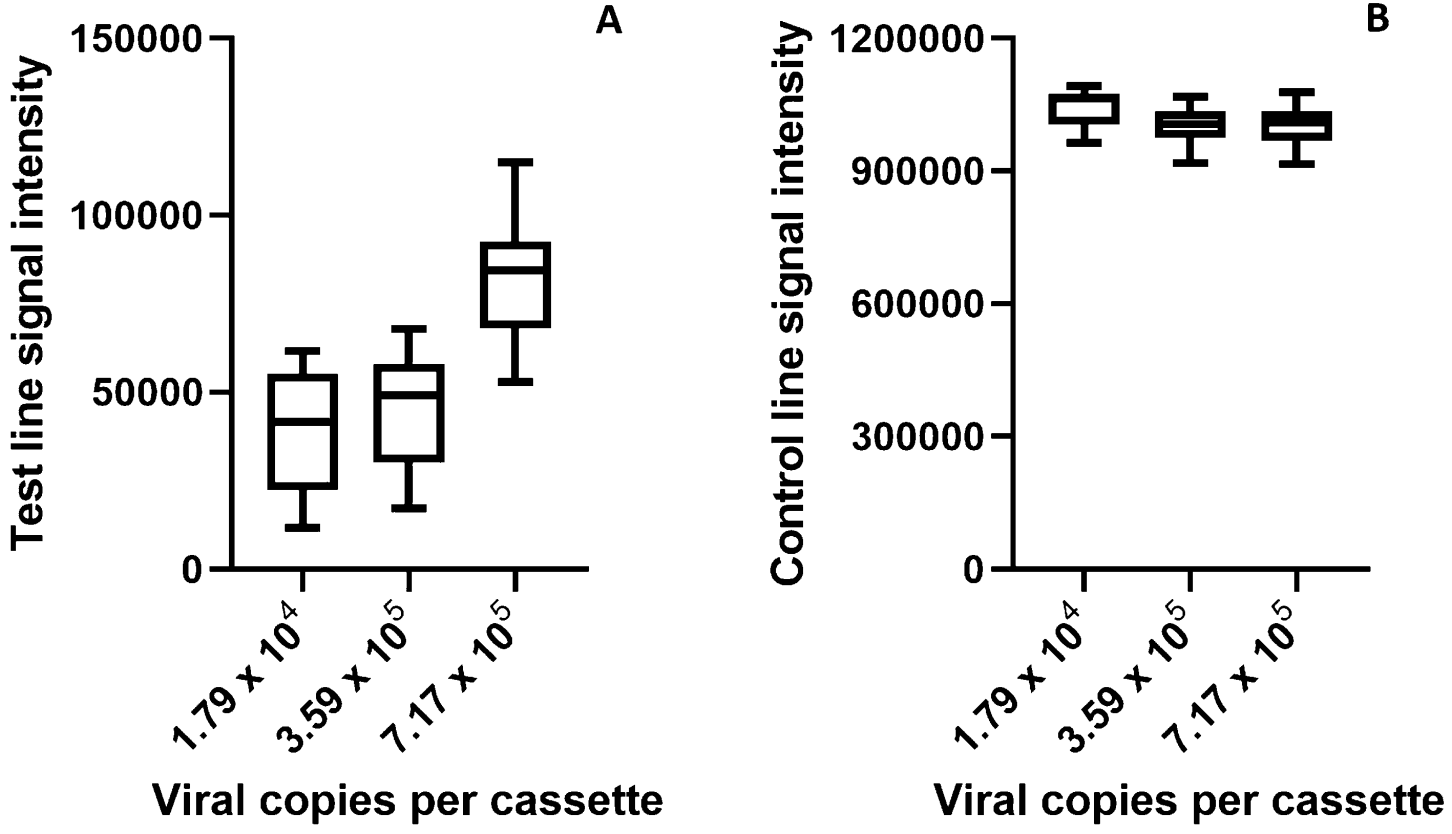
Test line signal intensity on the SARS-CoV-2 RDT cassettes were measured using the RDS-2500 reader (**A**). Samples at 1.79 × 10^4^ viral genomic copies/RDT samples, visually below the LOD, have the lowest signals at the test line location with a median test line signal of 38,750. Samples at the visual LOD of 3.59 × 10^4^ viral copies/RDT have higher signal with a median test line signal of 43,658. Samples above the visual LOD at 7.17 × 10^4^ viral copies/RDT have significantly higher signal with a median test line signal of 81,924. For all 50 cassettes tested, the control line intensity was strongly positive, indicating all tests were valid (**B**). Samples with more viral antigen present (≥ 3.59 × 10^4^ viral copies/RDT) showed a control line signal approximately 5% lower than cassettes where less or no analyte was present, however this was not discernable by visual inspection.

To quantitatively assess the sensitivity of the SARS-CoV-2 antigen RDT, each of the fifty cassettes used in the determination of the assay LOD were read using the RDS-2500 reader. Figure 5 demonstrates the range of signals detected at the test line and the control line locations.

**Figure 6:**
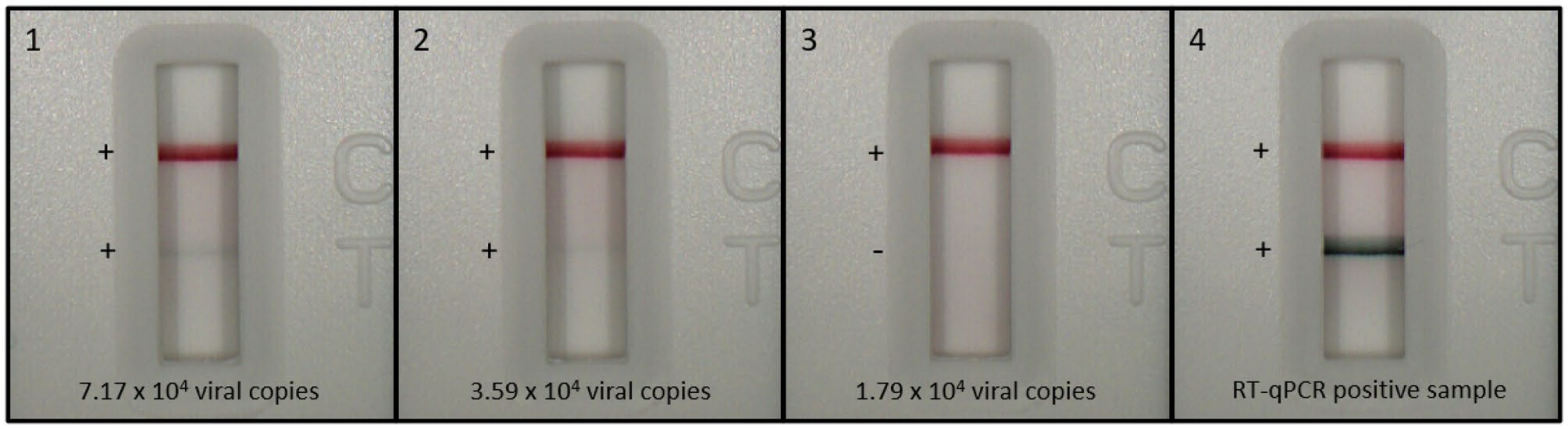
Representative RDT cassettes of each level of inactivated virus-spiked negative nasal swab samples at or around the LOD alongside an RT-qPCR positive swab sample. Weak positive blue test lines are visible in the “T” location of cassettes 1 and 2, both at and above 3.59 × 10^4^ viral copies/RDT. No test line is visible on cassette 3, below 3.59 × 10^4^ viral copies/RDT. For comparison, the RT-qPCR positive swab sample demonstrates a clearly visible test line on cassette 4 from a highly positive patient sample. Control lines are visible at location “C” for all cassettes 1–4.

The reader can interpret signal outside of the actual band, which increases the perceived background signal (as shown by the overlapping error bars of the 1.79 × 10^4^ viral genomic copies and the 3.59 × 10^4^ viral copies in Fig. 5A). Visual images of representative RDTs are therefore shown in Fig. 6 to highlight the intensity level of the band for each level of inactivated virus-spiked negative nasal swab samples alongside a RT-qPCR positive swab sample. Weak positive blue test lines were visible in the “T” location of cassettes 1 and 2, both at and above 3.59 × 10^4^ viral copies/RDT. No test line was visible on cassette 3, below 3.59 × 10^4^ viral copies/RDT. For a comparison, the RT-qPCR positive swab sample demonstrates a clearly visible test line on cassette 4. Control lines were visible at location “C” for all cassettes 1–4.

### Clinical sample performance testing

Clinical sample performance testing as outlined by the FDA EUA recommendation is a minimum of 30 positive specimens and 30 negative specimens collected either retrospectively or prospectively. Tests should demonstrate a minimum sensitivity of ≥ 80% for sample types tested^14^. Retrospective nasal samples collected into saline were mixed with 10X extraction buffer to mimic our intended sample-extraction buffer concentration and run on RDT cassettes; all samples were tested for SARS-CoV-2 by RT-qPCR using the ExoDx COVID-19 RT-qPCR Test (a CLIA-validated FDA EUA-authorized CDC protocol; this assay has an LOD of ≤ 0.85 copies/µL). Samples are considered positive by RT-qPCR if both the N1 and N2 assay are detected. Cycle threshold (Ct) levels are inversely proportional to the amount of viral nucleic acid in the sample. A dose–response curve of Ct values vs SARS-CoV-2 RNA copies using the clinically validated RT-qPCR assay is shown in Supplemental Fig. 2.

**Figure 7:**
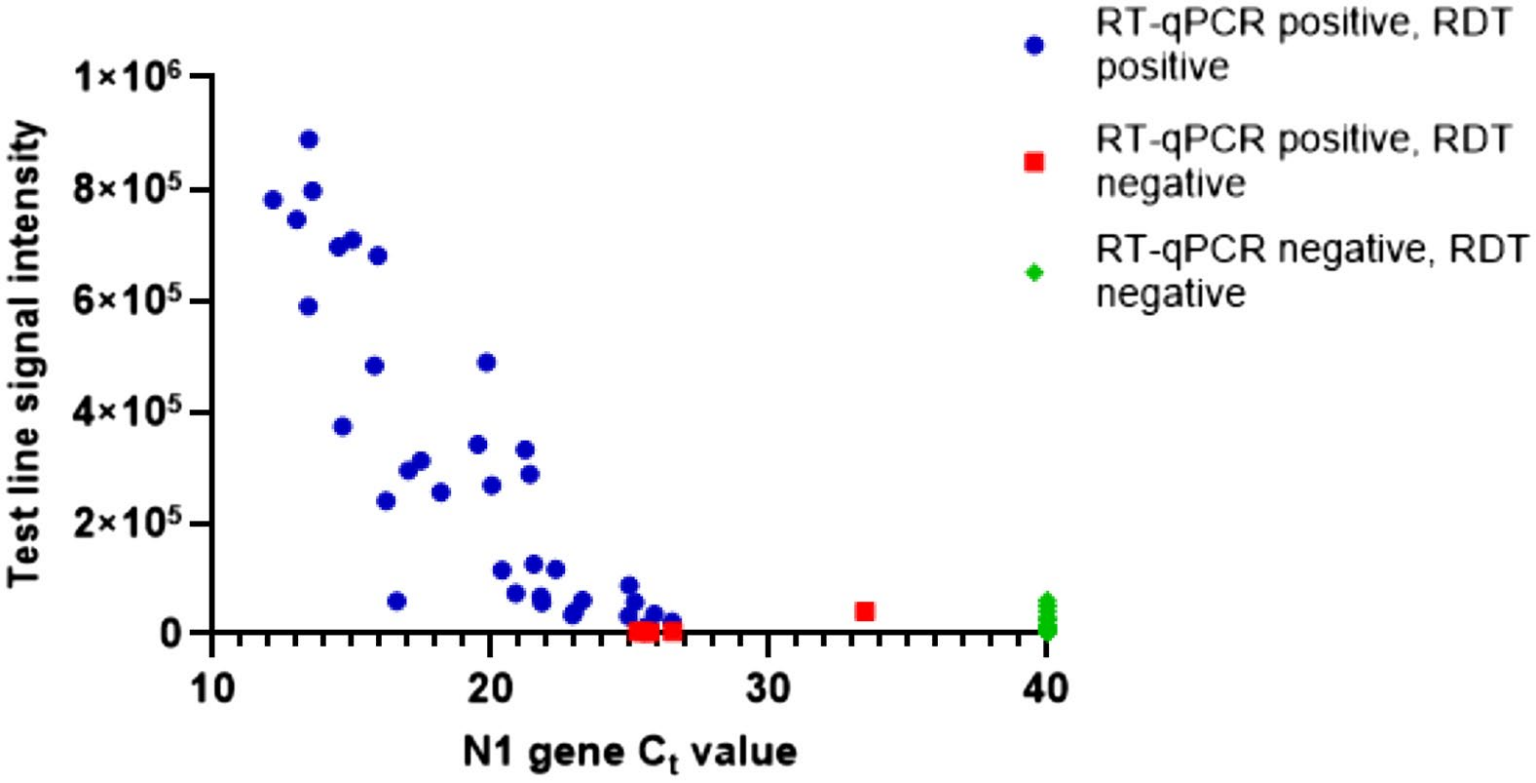
Correlation of SARS-CoV-2 RDT signal intensity to RT-qPCR N1 Ct in patient nasal swabs. SARS-CoV-2 RT-qPCR positive samples that were positive by RDT are shown in blue circles, the majority being ≤ 25 Ct with the highest at 26.5 Ct. RT-qPCR positive samples that were negative by RDT are shown in red squares and are above 25 Ct. SARS-CoV-2 RT-qPCR negative samples are shown in green diamonds, all have low signal intensity levels at the test line location and therefore are RDT-negative.

A total of 35 out of 40 nasal swab samples positive by RT-qPCR were shown positive by RDT, demonstrating 87.5% sensitivity, and 40 out of 40 patient nasal swab samples negative by RT-qPCR were negative by RDT, demonstrating 100% specificity. This performance from a preliminary evaluation is comparable to SARS-CoV-2 RDT devices that have received FDA EUA approval^15^. However, it is important to note that the reported clinical sensitivity of these assays is dependent on the viral load of the patients in the clinical cohort. The viral load varies widely between patients and is dependent on when and how the sample was taken during the course of an infection, making it challenging to compare across different studies. The concordance between RDT and RT-qPCR testing of patient nasal swabs collected into saline is summarized in Table 3 and Fig. 7; data for each individual test cassette is listed in Supplemental Table 1 and individual cassette images are shown in Supplemental Fig. 3.

**Table 3:** Concordance of the SARS-COV-2 antigen RDT assay and the ExoDx COVID-19 RT-qPCR assay.

RT-qPCR result	RDT test line result	RDT control line result
Negative	0/40	40/40
Positive	35/40	40/40

SARS-CoV-2 antigen RDT results achieved 87.5% sensitivity and 100% specificity in a clinical cohort of 40 negative samples and 40 positives that were confirmed by RT-qPCR.

SARS-CoV-2 RT-qPCR-positive samples that had Ct values of 25 or lower were universally positive in the SARS-CoV-2 antigen RDT. RT-qPCR positive samples that were not detected as positive by RDT had Ct values of 25.3, 25.5, 25.7, 26.5 and 33.5, respectively, for the N1 assay. This indicates that the LOD for the SARS-CoV-2 antigen RDT assay correlates with patient samples that have Ct values around 25 for this CLIA-validated FDA EUA-authorized CDC test protocol.

**Figure 8:**
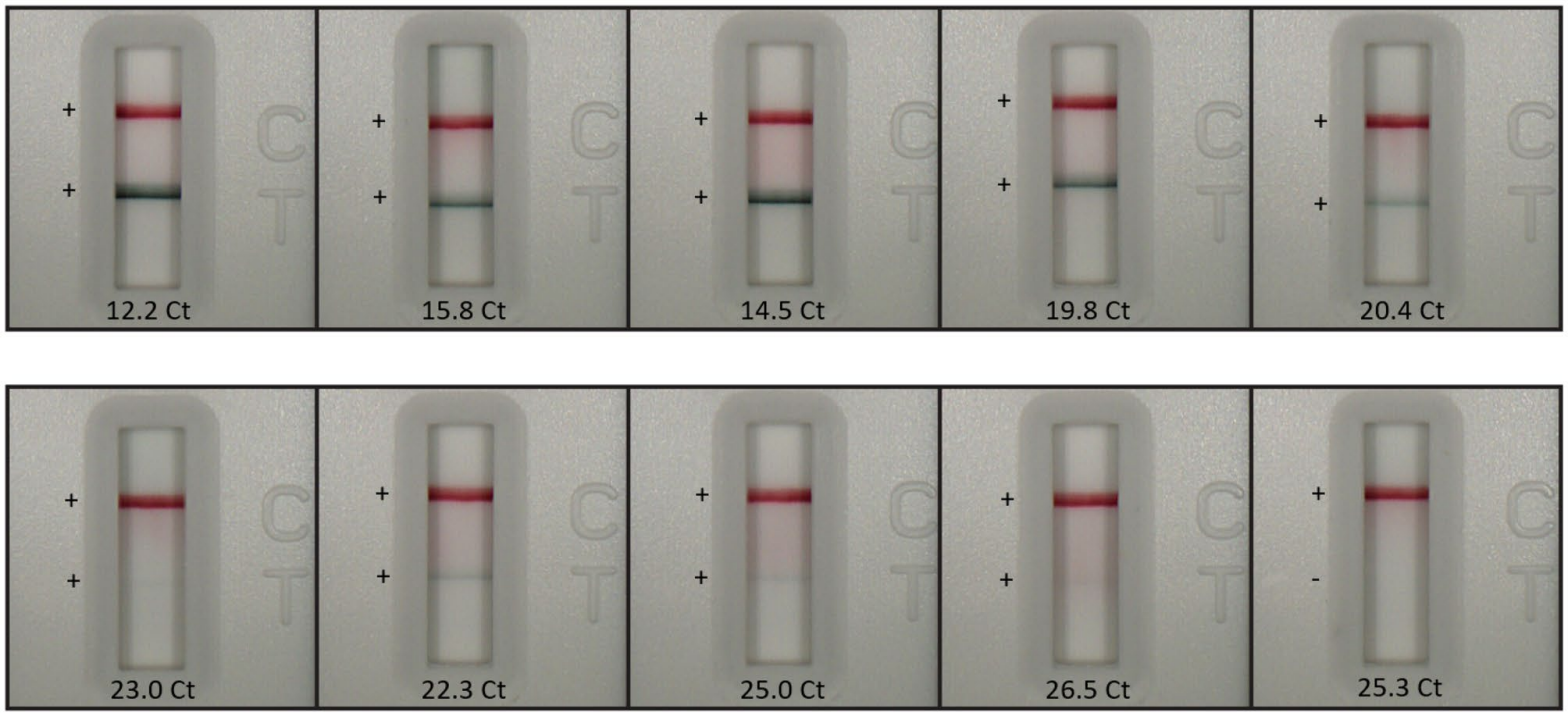
Representative test images of test kit-based study. Visual inspection of SARS-CoV-2 RDT cassettes with different viral load shows diminishing test line signal with decreasing amount of applied virus. Images of RT-qPCR known positive SARS-CoV-2 saline swab samples were collected 15 min from sample application; 400 µL of saline swab sample was added to sample extraction buffer tube and evaluated on SARS-CoV-2 antigen RDT. N1 Ct value for each sample is depicted on its corresponding image.

To further assess the SARS-CoV-2 antigen RDT kit components and determine if a higher dilution of the samples could be used (using the 1X extraction buffer instead of 10X), 10 additional samples were evaluated by combining 400 µL of saline-collected swab directly into the kit sample tube containing 400 µL of extraction buffer, pipetting up and down to mix, and adding 3 drops (approximately 85 µL) to the sample port of the RDT cassette. As was observed previously, all known positive RT-qPCR samples with Ct values ≤ 25.0 resulted in a positive test line on the RDT; one sample at 26.5 Ct reported positive on RDT while one sample at 25.28 Ct was negative. Visual results are depicted in Table 4 and Fig. 8.

**Table 4:** Visual interpretation summary of test kit-based study.

RT-qPCR N1 Ct	Test line result (+/−)	Control line result (+/−)
12.2	+	+
15.8	+	+
14.5	+	+
19.8	+	+
20.4	+	+
23.0	+	+
22.3	+	+
25.0	+	+
26.5	+	+
25.3	** − **	** + **

400 µL of RT-qPCR known positive SARS-CoV-2 saline swab samples added to sample extraction buffer tube and evaluated on SARS-CoV-2 antigen RDT. The results agree with previous cutoff for RT-qPCR positives on RDT around 25 Ct for the N1 assay.

## Discussion

We have described the development of a SARS-CoV-2 antigen RDT with preliminary performance data that meets acceptance criteria for sensitivity and specificity as outlined by the FDA in their EUA guidance documents and is among the most sensitive lateral flow assays that do not require a reader. A summary of other Emergency Use Authorization (EUA) issued antigen tests for SARS-CoV-2 collected in October of 2020 from the Food and Drug Administration (FDA) website demonstrated a range of LOD from 1.0 × 10^2^ TCID_50_/mL to 4.5 × 10^5^ TCID_50_/mL^15^. Our LOD of 3.59 × 10^4^ viral copies per test is equivalent to 2.0 × 10^2^ TCID_50_/mL and therefore is among the most sensitive SARS-CoV-2 RDT devices. For comparison, the BinaxNOW COVID-19 Ag card has shown a limit of detection of about 4.04 × 10^4^ to 8.06 × 10^4^ copies per swab^16^. Additional studies to examine cross-reactivity with other infectious viral and bacterial species, potential interference from common endogenous substances, and additional clinical performance testing with prospectively collected samples should be performed to fully establish the performance of the test.

All the current SARS-CoV-2 antigen RDTs have sensitivity disadvantages compared to the gold standard RT-qPCR. However, the fact that RDTs can be more widely distributed, are cheaper, do not require an instrument or a reader, and can be used without specialized training is a big advantage. A test that is less sensitive but more readily available may identify more cases than a more sensitive test that is less available. To enable efficient surveillance of COVID-19 and reduce community transmission, quick and inexpensive testing is required. The longer turn-around time for RT-qPCR (in many cases several days) also makes it less useful. Patients with lower virus levels not detected by RDT may be in a later phase of a waning infection, or they may be early in disease course and becoming more infectious over time. While RDTs have a lower analytical sensitivity than RT-qPCR, the ability to pick up community spread may be higher. RT-qPCR was estimated to detect at best 10% of infections in the US, and this number is likely far lower in countries with less access to these types of tests^8^. These RDTs enable immediate results, ease of use, accessibility, and no need for sophisticated equipment. They can also be easily mass-produced, and tests that utilize different supply chains and reagents are needed when global demand for testing occur as was seen during this pandemic.

Furthermore, RT-qPCR tests are not immune to potential false negatives as new SARS-CoV-2 variants pose an evolving challenge to global testing. The variants B.1.1.7 first identified in the UK^17^, P.1 first identified in Brazil^18^, and variant B.1.351 first identified in South Africa^19^, have drawn concern that laboratories performing RT-qPCR tests targeting the gene for the viral spike protein alone may have decreased sensitivity^20^. Our SARS-CoV-2 antigen RDT, as well as many other RDTs, targets the nucleocapsid protein. As of early February 2021, the Foundation for Innovative New Diagnostics has examined 5 of the most widely used SARS-CoV-2 RDTs and determined these 3 viral variants had minimal anticipated impact on performance^21^. Regardless, all SARS-CoV-2 test manufacturers must monitor globally emerging mutations, including mutations in genes coding for the nucleocapsid protein, for all diagnostic applications^22^.

It is critical that diagnostic innovation reach all populations globally to enhance surveillance and monitor viral transmission—and RDTs can play an important role in quenching this SARS-CoV-2 pandemic when used appropriately to help alleviate testing inequality. Settings where centralized laboratory tests are limited can use RDTs as a complement to RT-qPCR, and high-risk congregate settings can use frequent serial screening tests to quickly identify SARS-CoV-2 infection and inform control measures to limit transmission, especially in settings where RT-PCR testing is not available. A pandemic of this size and scope that has reached all corners of the globe will require a diagnostic approach that matches its scale, and SARS-CoV-2 antigen rapid diagnostic tests are a crucial tool in the arsenal we must gather towards this effort.

## Supplementary Information

Supplementary Information 1.
