# Supplementary material for: A SARS-CoV-2 antigen rapid diagnostic test for resource limited settings
Source: Sci Rep. 2021 Nov 26;11:23009. doi: 10.1038/s41598-021-02128-y (PMC8626481; doi:10.1038/s41598-021-02128-y)
Supplement: Supplementary file 1 — Supplementary Information 1. [file 41598_2021_2128_MOESM1_ESM.pdf]

Supplemental Figures:

Supplemental Figure 1. Images of each RDT test cassette result for LOD robustness study. Images 1-20 are test cassettes run with 7.17×10^4 viral copies/RDT, images 21-40 are test cassettes run with 3.59×10^4 viral copies/RDT, and images 41-50 are test cassettes run with 1.79×10^4 viral copies/RDT.

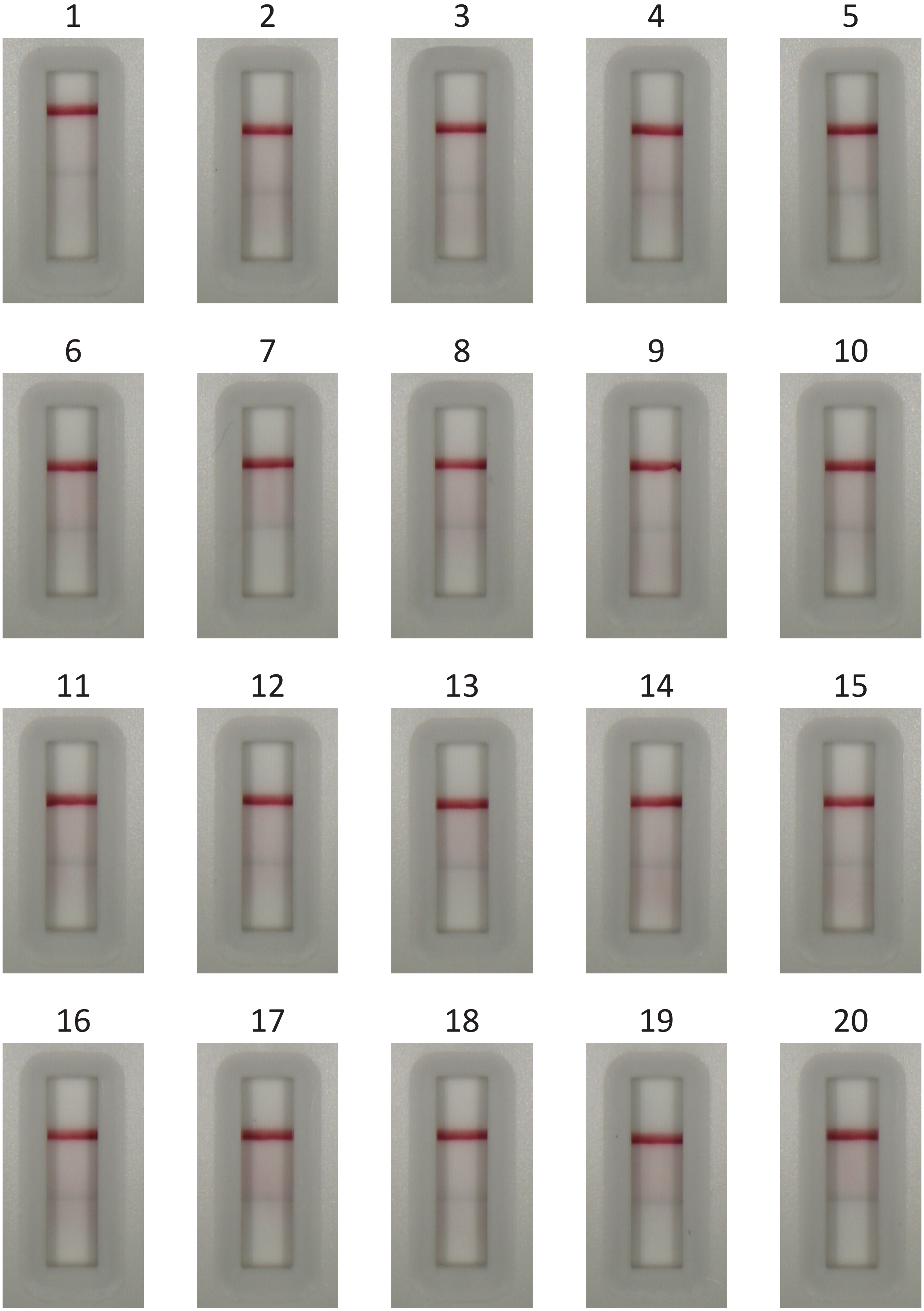

21

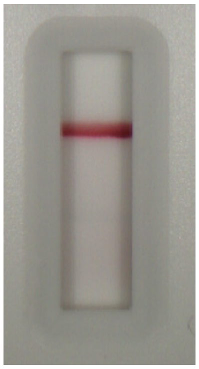

22

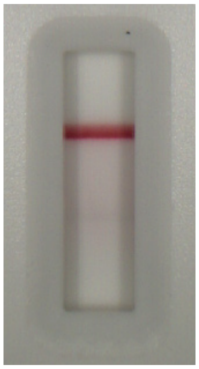

23

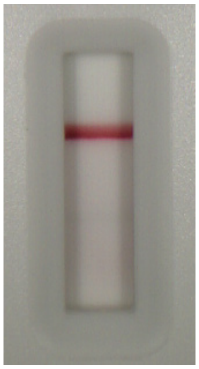

24

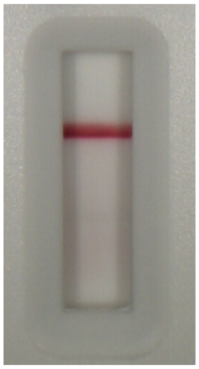

25

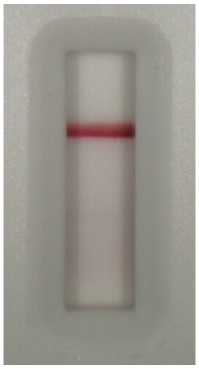

26

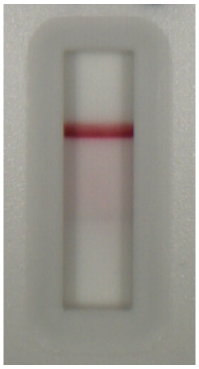

27

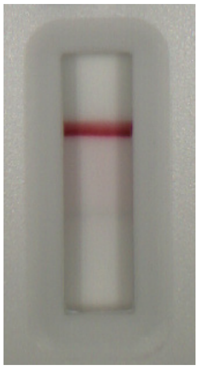

28

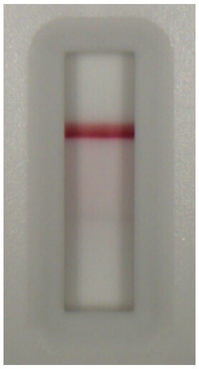

29

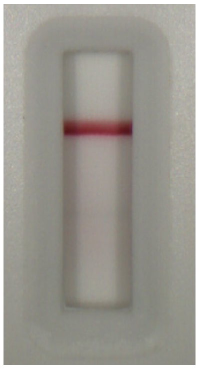

30

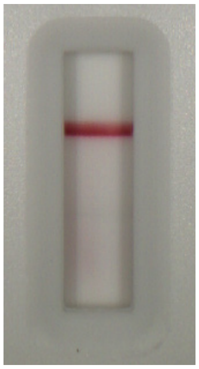

31

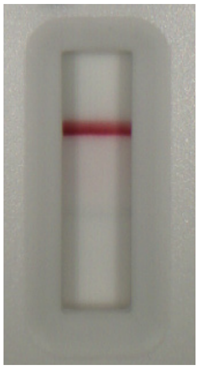

32

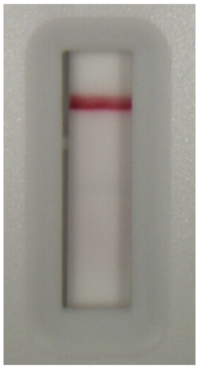

33

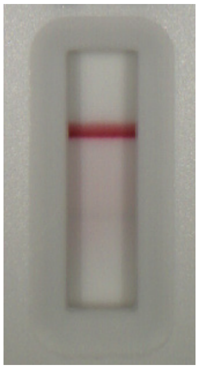

34

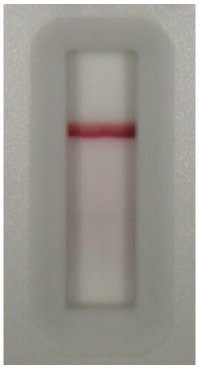

35

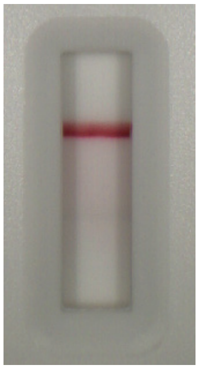

36

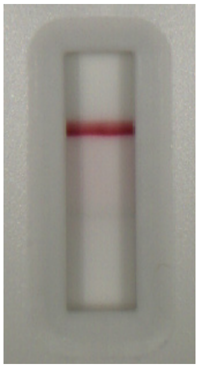

37

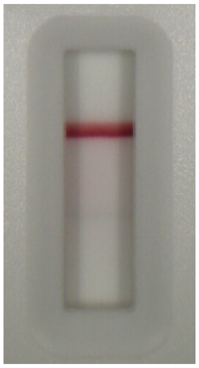

38

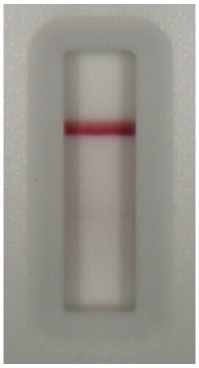

39

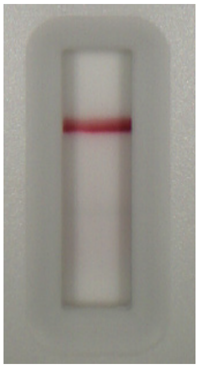

40

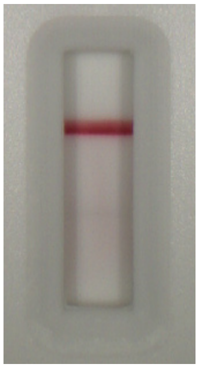

41

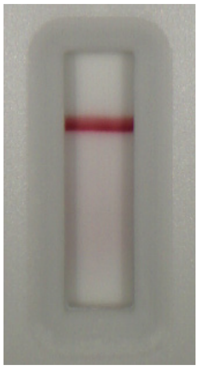

42

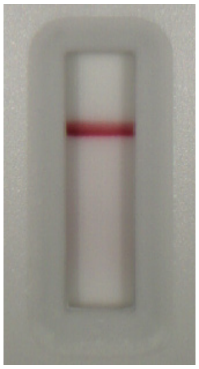

43

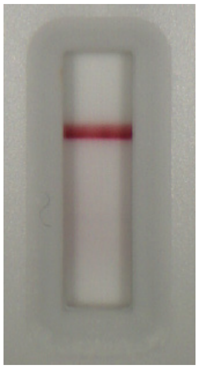

44

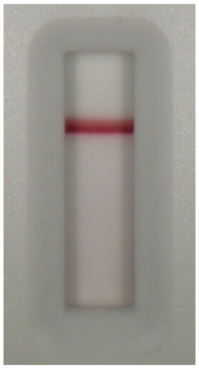

45

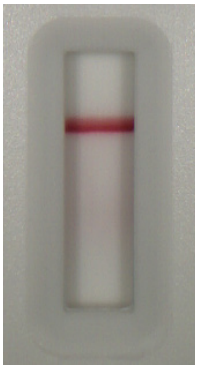

46

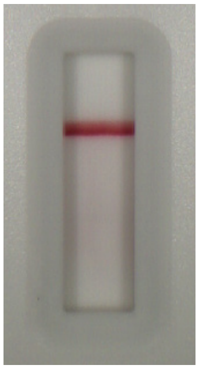

47

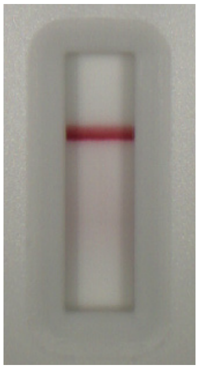

48

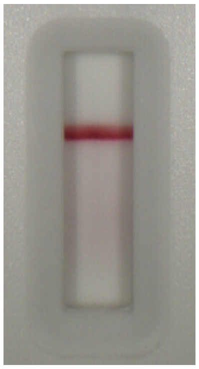

49

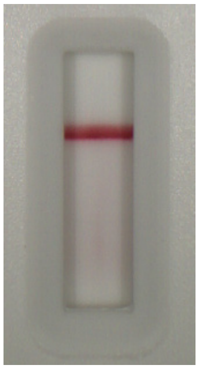

50

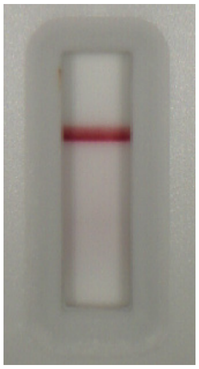

Supplemental Figure 2. qRT-PCR Ct values as a function of SARS-CoV-2 RNA genome reference material. The virus copy number on the x-axis is adjusted for the volume used in a RDT cassette where 10% of the volume is extraction buffer. This indicate that the LOD (virus genome copies) on clinical samples is very similar to the heat-inactivated SARS-CoV-2 reference material, where the lateral flow RDT assay LOD is around 35-40,000 RNA copies per cassette

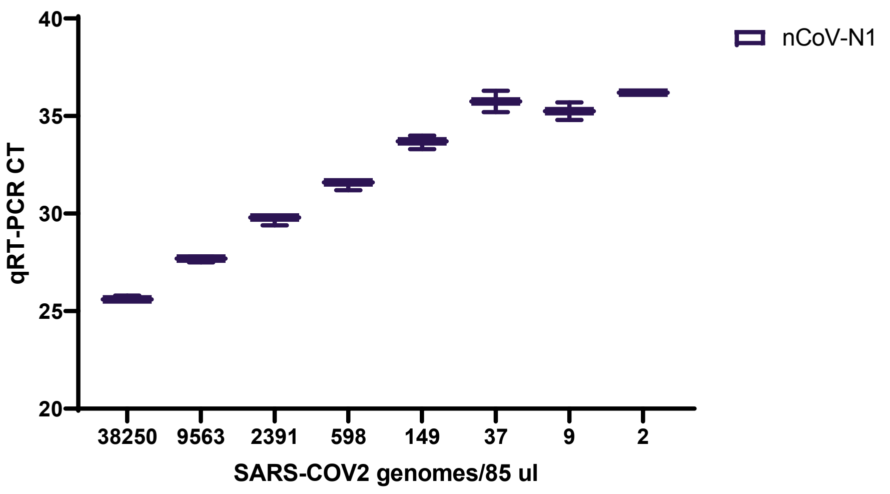

.

Supplemental Figure 3. Images of each RDT test cassette result for clinical sample performance testing. Images 1-40 are test cassettes run with patient nasal swab samples confirmed negative by RT-qPCR. Images 41-80 are test cassettes run with patient nasal swab samples confirmed positive by RT-qPCR. Negative clinical samples 2, 14, 23, 25, 34, 37 and 40 had elevated test line assay signal intensity by RDS-2500 reader ranging from 24498 to 60720 counts caused by slow flowing red colored control particles. No blue test line was visible for these test cassettes; they are determined visually as negatives. Weak positive clinical samples44, 46, 47, 48, 49, 52, 57, 59, 72, 73, and 75 had low test line assay signal intensity by RDS-2500 reader ranging from 12704 to 60148 counts. Weak blue colored test line was visible for these test cassettes; they are determined visually as positives.

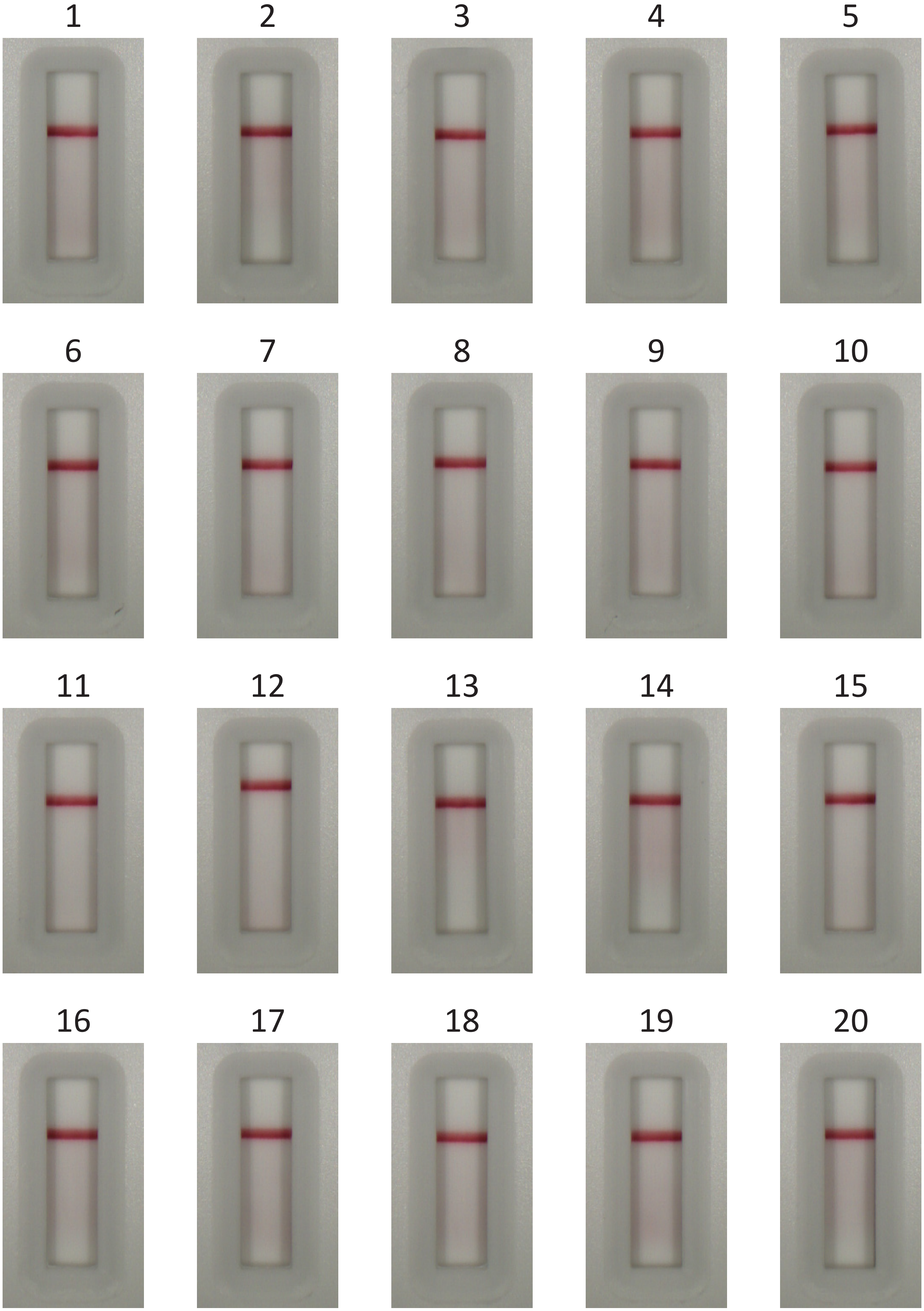

21 22 23 24 25

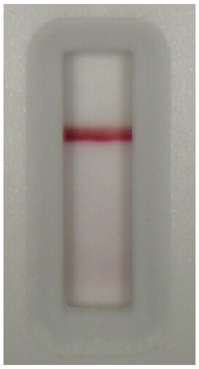
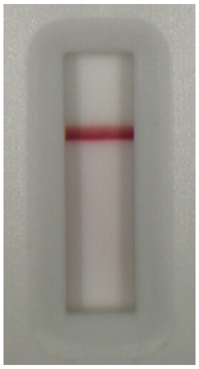
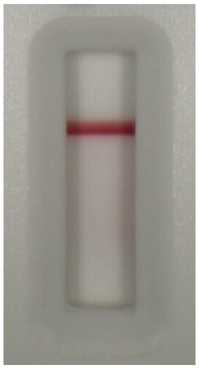
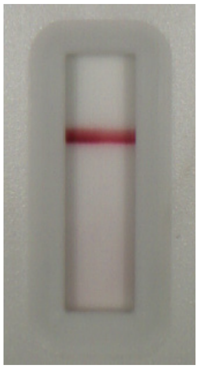
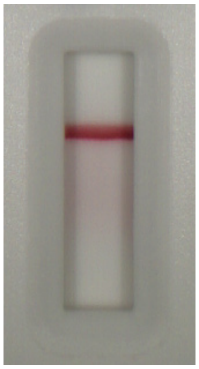

26 27 28 29 30

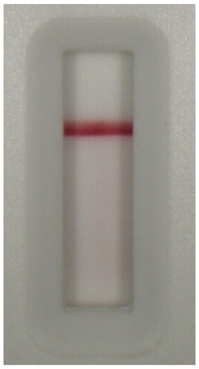
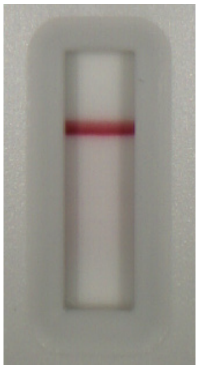
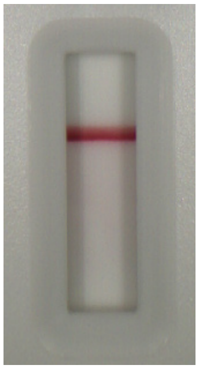
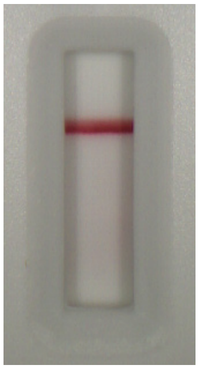
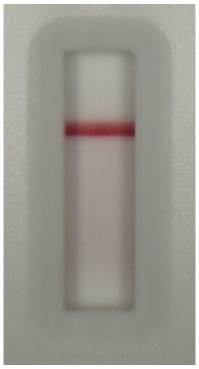

31 32 33 34 35

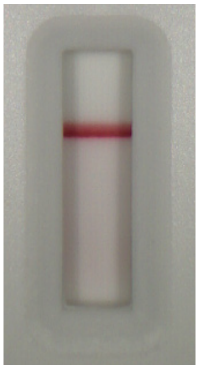
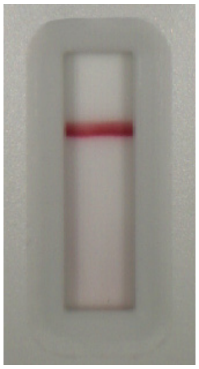
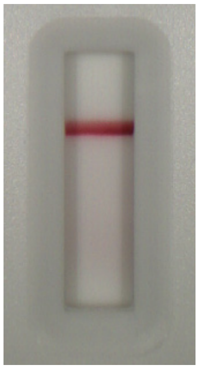
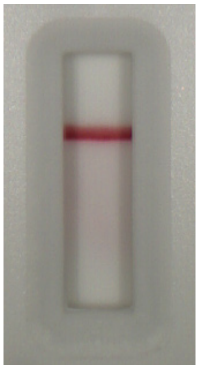
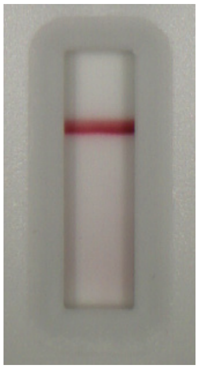

36 37 38 39 40

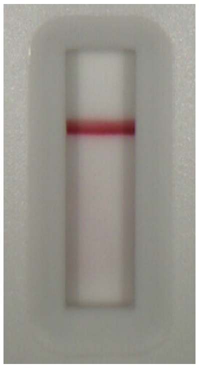
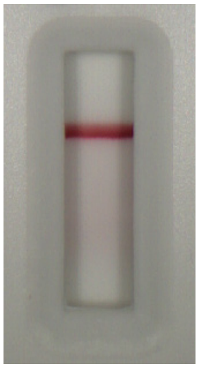
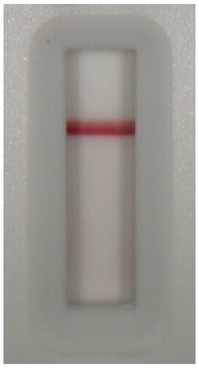
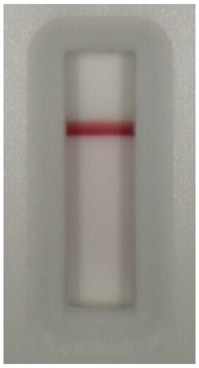
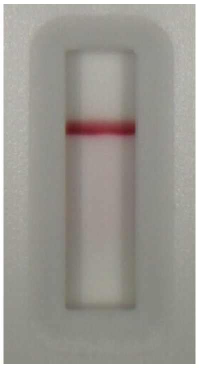

41

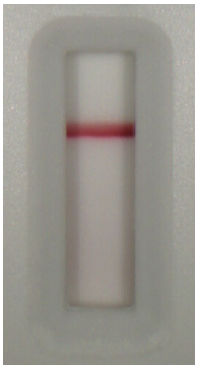

42

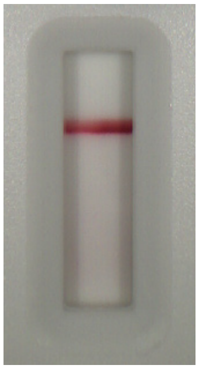

43

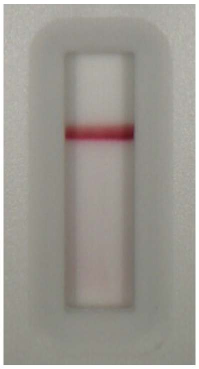

44

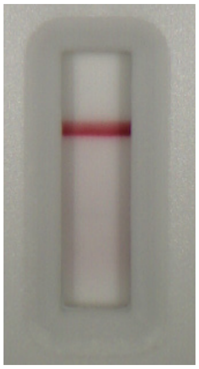

45

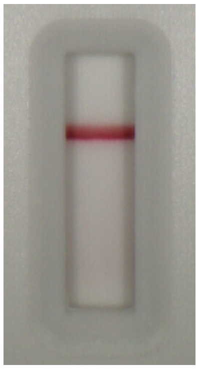

46

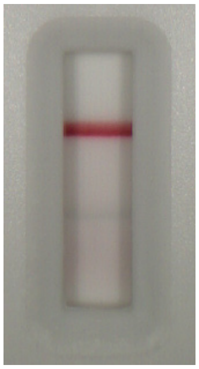

47

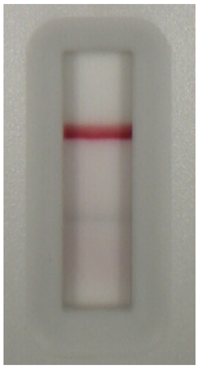

48

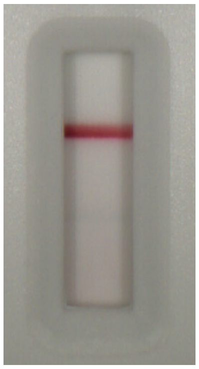

49

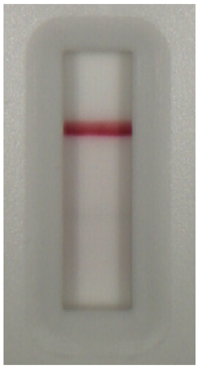

50

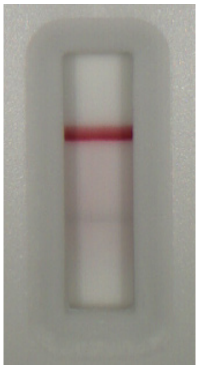

51

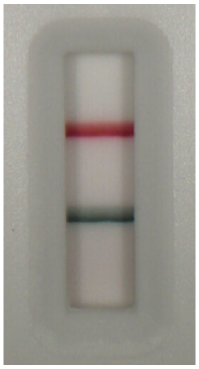

52

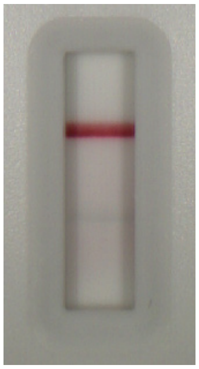

53

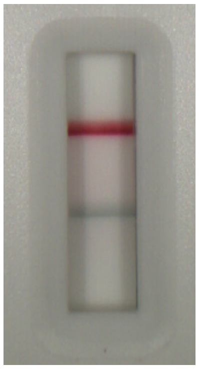

54

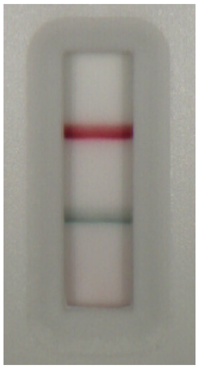

55

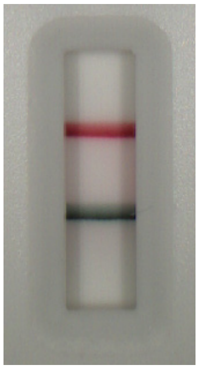

56

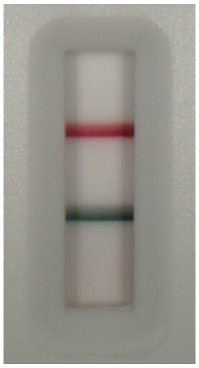

57

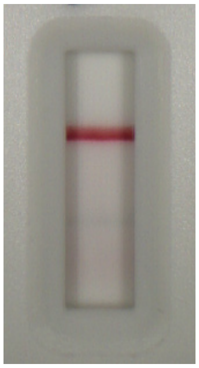

58

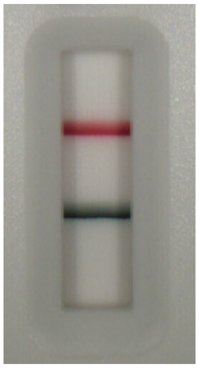

59

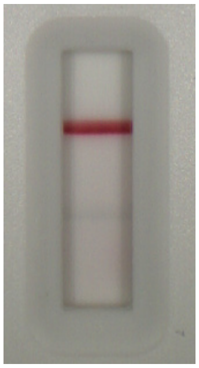

60

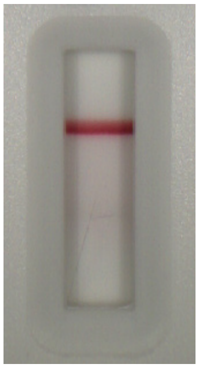

61 62 63 64 65

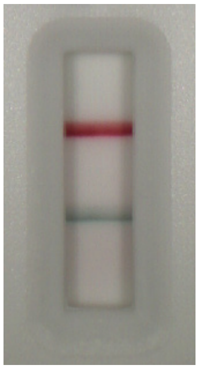 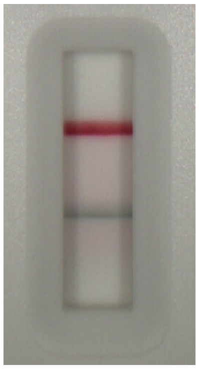 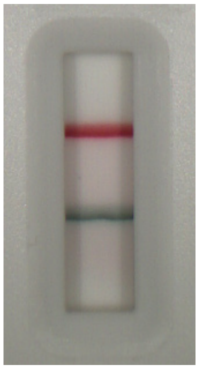 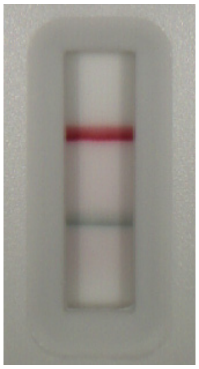 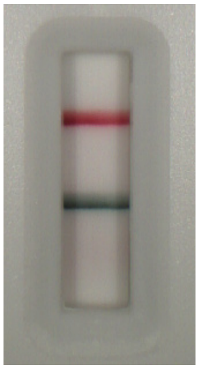

66 67 68 69 70

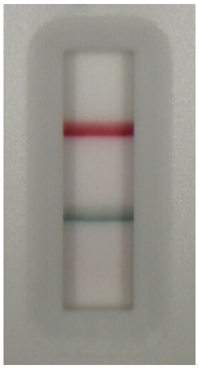 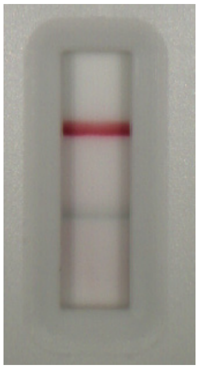 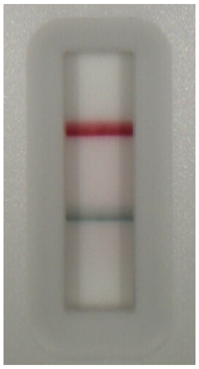 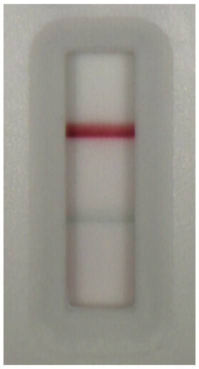 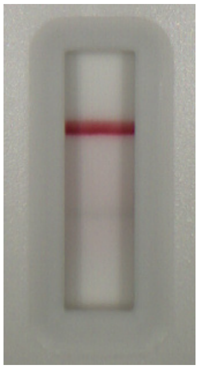

71 72 73 74 75

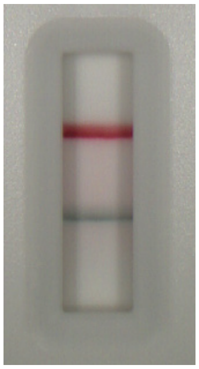 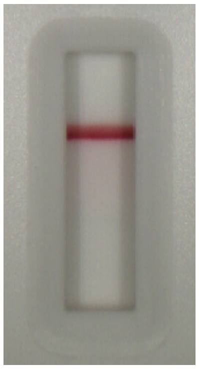 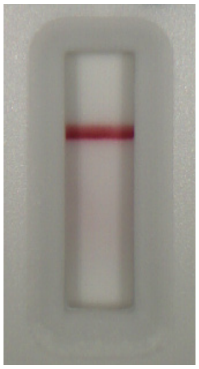 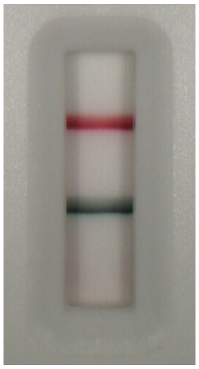 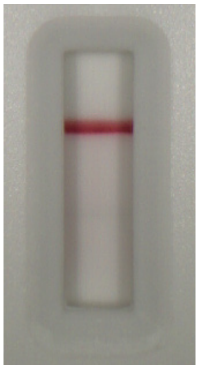

76 77 78 79 80

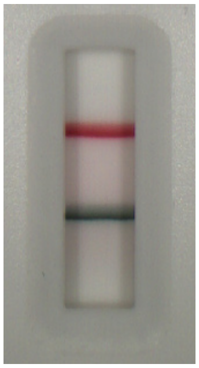 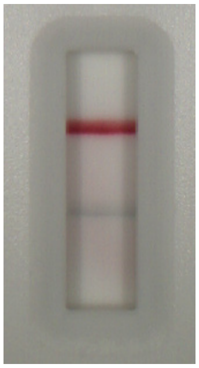 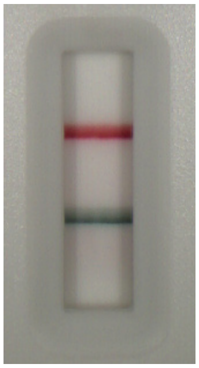 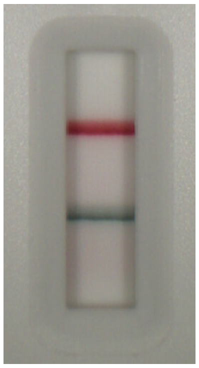 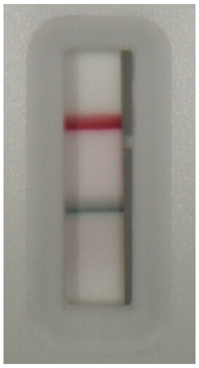

Supplemental Table 1: Concordance of the SARS-COV-2 antigen RDT assay and a validated RT-qPCR assay. Sample data points for anterior nares samples are listed by sample number, symptomatic status, qPCR result, N1 and N2 Ct (separate qPCR assays for the N gene), RP Ct (RNAse P for qPCR assay internal control) RDT test line intensity, RDT control line intensity, and visual result interpretation on the RDT cassette.

Sample #	Symptomatic	qPCR result	N1 Ct	N2 Ct	RP Ct	Test line intensity	Control line intensity	Visual Result
1	No	Negative			20.115	2453	1063677	Negative
2	No	Negative			25.745	24498	1040337	Negative
3	No	Negative			21.471	10795	1061696	Negative
4	Unknown	Negative			15.351	11568	1009354	Negative
5	No	Negative			22.329	7738	1041278	Negative
6	Yes	Negative			21.393	5389	1074842	Negative
7	No	Negative			23.099	7780	1053191	Negative
8	No	Negative			24.585	8750	1008736	Negative
9	Yes	Negative			23.202	5467	1014812	Negative
10	Yes	Negative			24.682	5082	961988	Negative
11	No	Negative			21.501	4813	1000495	Negative
12	No	Negative			23.483	3587	1074691	Negative
13	No	Negative			27.292	6433	1029907	Negative
14	No	Negative			24.656	39512	1014964	Negative
15	No	Negative			27.693	8972	1088044	Negative
16	No	Negative			27.354	14924	1018827	Negative
17	No	Negative			24.574	7235	994401	Negative
18	No	Negative			24.385	7454	1016481	Negative
19	No	Negative			25.348	9013	1077262	Negative
20	No	Negative			28.175	13300	1001479	Negative
21	No	Negative			26.319	11447	1017075	Negative
22	No	Negative			24.611	8851	1023499	Negative
23	No	Negative			29.731	52183	1032756	Negative
24	No	Negative			24.16	12410	1000537	Negative
25	No	Negative			30.218	29900	1027507	Negative
26	No	Negative			25.178	6103	1008776	Negative
27	No	Negative			25.553	10370	980612	Negative
28	No	Negative			24.164	11765	1019498	Negative
29	No	Negative			20.553	8252	1070557	Negative
30	No	Negative			27.851	9948	956342	Negative
31	No	Negative			23.181	4699	1032988	Negative
32	No	Negative			24.858	5292	1075290	Negative
33	No	Negative			20.410	10692	1058029	Negative
34	No	Negative			25.741	48430	1008208	Negative
35	Yes	Negative			25.078	6459	1060540	Negative
36	No	Negative			24.972	5119	1021698	Negative
37	No	Negative			25.921	60720	1029226	Negative
38	Yes	Negative			22.561	5653	1000954	Negative
39	No	Negative			27.545	13160	1053343	Negative
40	No	Negative			23.579	24990	1037042	Negative

Sample #	Syptomatic	qPCR result	N1 Ct	N2 Ct	RP Ct	Test line intensity	Control line intensity	Visual Result
41	No	Positive	25.49	25.98	23.68	3429	990644	Negative
42	No	Positive	26.54	27.21	23.87	3719	1037311	Negative
43	No	Positive	25.7	26.65	19.44	3128	1041137	Negative
44	Yes	Positive	26.5	26.33	29.24	22013	995537	Positive
45	No	Positive	25.28	24.19	23.42	3478	1053294	Negative
46	Yes	Positive	25.18	26.79	22.97	56864	1020904	Positive
47	Yes	Positive	23.29	22.95	22.92	60148	1011727	Positive
48	Yes	Positive	24.94	27.08	24.43	32034	1013268	Positive
49	Yes	Positive	25.88	25.92	25.79	35411	1080769	Positive
50	Yes	Positive	24.97	24.59	24.97	86907	1024989	Positive
51	Unknown	Positive	15	14.49	21.37	708209	1098597	Positive
52	No	Positive	16.61	15.96	27.23	58970	989820	Positive
53	No	Positive	16.22	14.92	23.92	238541	1025565	Positive
54	Unknown	Positive	17.48	16.78	24.18	311517	1071887	Positive
55	Unknown	Positive	13.57	13.34	20.67	796461	1063039	Positive
56	Unknown	Positive	13.01	12.19	22.19	744276	1060009	Positive
57	No	Positive	22.93	21.92	22.33	34023	958220	Positive
58	Unknown	Positive	13.45	13.16	24.44	888951	1087535	Positive
59	No	Positive	21.82	22.28	23.73	55946	1094307	Positive
60	Yes	Positive	20.89	20.63	22.89	72549	1033483	Positive
61	Yes	Positive	17.03	17.01	25.22	294025	1085063	Positive
62	No	Positive	20.02	20.22	23.51	266810	1074262	Positive
63	No	Positive	15.8	15.31	24.6	482173	1038514	Positive
64	No	Positive	18.19	17.91	25.19	254225	1057494	Positive
65	Yes	Positive	14.51	13.52	22.97	695520	1028622	Positive
66	No	Positive	14.65	13.53	23.21	373031	1028774	Positive
67	Yes	Positive	20.39	19.82	22.44	114588	1010560	Positive
68	Yes	Positive	21.22	20.99	23.4	330943	1080957	Positive
69	Unknown	Positive	22.32	22.28	23.45	116251	1102317	Positive
70	No	Positive	21.79	21.32	24.87	67432	1025029	Positive
71	Yes	Positive	21.39	21.46	24.65	286729	925170	Positive
72	Yes	Positive	25.56	26.98	25.98	12704	1044763	Positive
73	Yes	Positive	33.46	31.87	26.52	40004	1052656	Negative
74	Yes	Positive	12.15	10.9	22.79	780660	1057401	Positive
75	Yes	Positive	22.99	22.19	24.83	39519	1020148	Positive
76	Yes	Positive	15.93	14.87	26.39	679697	1017169	Positive
77	Yes	Positive	21.54	21.73	25.83	125574	1062806	Positive
78	Yes	Positive	13.43	12.18	22.78	588649	1041460	Positive
79	Yes	Positive	19.84	18.98	22.81	487732	1098439	Positive
80	Yes	Positive	19.52	18.75	23.74	340446	937063	Positive
